# Sensory specific satiety: More than ‘just’ habituation?

**DOI:** 10.1016/j.appet.2016.04.019

**Published:** 2016-08-01

**Authors:** Laura L. Wilkinson, Jeffrey M. Brunstrom

**Affiliations:** a Department of Psychology, Swansea University, Singleton Park, Swansea, SA2 8PP, UK; b Nutrition and Behaviour Unit, School of Experimental Psychology, University of Bristol, 12a Priory Road, Bristol, BS8 1TU, UK

**Keywords:** Sensory specific satiety, Commodity theory, Habituation, Perceived variety

## Abstract

Sensory specific satiety (SSS) describes the decline in pleasantness associated with a food as it is eaten relative to a food that has not been eaten (the ‘eaten’ and ‘uneaten’ foods, respectively). The prevailing view is that SSS is governed by habituation. Nevertheless, the extent to which SSS results solely from this ‘low-level’ process remains unclear. Three experiments were conducted to explore the hypothesis that ‘top-down’ cognitive activity affects the expression of SSS; specifically, we manipulated participants' expectations about whether or not they would have access to alternative test foods (uneaten foods) after consuming a test meal (eaten food). This manipulation was motivated by ‘Commodity Theory,’ which describes the relative increase in value of a commodity when it becomes unavailable. We tested the hypothesis that a decline in the pleasantness and desire to eat the eaten food is exaggerated when uneaten foods are unavailable to participants. None of our findings supported this proposition – we found no evidence that SSS is dependent on top-down processes associated with the availability of other uneaten test foods.

## 1 Introduction

The term ‘sensory specific satiety’ (SSS) relates to the decline in pleasantness of a food as it is eaten relative to ‘uneaten’ foods that have different sensory qualities (Rolls, Rolls, Rowe, & Sweeney, 1981). SSS is thought to promote both the termination of an eating episode (Hetherington, 1996) and the tendency to resume eating when different foods are made available (e.g., desserts) (Rolls et al., 1981). These effects can be marked. For example, in one study comparing a single-course meal with a four-course meal, participants consumed 60% more in the multiple-course condition (Rolls, Van Duijvenvoorde, & Rolls, 1984).

Higgs, Williamson, Rotshtein, and Humphreys (2008) demonstrated SSS over multiple meals in amnesic patients. This suggests that SSS exists even in the absence of a ‘memory for recent eating’ and that it is governed by a mechanism that can operate outside of awareness. The authors suggest that this might be attributed to habituation (Higgs et al., 2008). However, it has also been suggested that SSS is governed by a more basic form of learning known as stimulus specificity (Havermans, 2012). Stimulus specificity is observed when a decline in responding occurs but subsequent dishabituation (when responding recovers upon presentation of an alternative stimulus) does not (Epstein et al., 2009, Havermans, 2012).

Other researchers have suggested that SSS might also be influenced by so called ‘top-down’ influences (Hetherington & Rolls, 1996), such as processing of context, motivation, and more general beliefs about a meal (Kinchla & Wolfe, 1979). In an early example, Rolls, Rowe, and Rolls (1982) offered participants chocolates that differed only in their colour and found that pleasantness of the eaten colour declined significantly more than pleasantness for the uneaten colours. Broadly consistent with this finding, other studies indicate that merely giving participants the perception that they are consuming a greater variety of flavoured test foods can delay satiation (Kahn and Wansink, 2004, Redden, 2008).

Havermans and Brondel (2013) explored how perceived variety might play a role in SSS. Participants were asked to taste and rate a set of four foods. They were then offered one of the foods to consume. By implication, the other uneaten foods were regarded as ‘unavailable variety’ at the beginning of the meal. However, in one condition the participants were told that they would be asked to examine a ticket halfway through their meal and that the ticket would determine whether they should continue with the same meal or switch to consume a previously unavailable food (in all cases a switch was never permitted). Relative to a standard control, this manipulation had little effect on SSS and on this basis Havermans and Brondel (2013) concluded that information about the potential availability of uneaten test foods has little impact on the development of SSS. On reflection, however, we note that at the half-way point in the test meal the availability of the uneaten foods was always resolved with certainty. Therefore, Haverman and Brondel (2013) approach relies on a manipulation to the potential availability of uneaten foods in the first half of a meal and the subsequent detection of this effect at the end of the meal. Since the second half of the meal was essentially identical in both conditions (participants were equally aware that the uneaten foods were unavailable) this paradigm may not be optimized to detect the specific phenomenon of interest.

The ‘decision making’ literature provides a theoretical framework within which to explore this idea. ‘Commodity Theory’ (Brock, 1968) describes the process that takes place when an individual evaluates a range of products that may or may not be available to them (Lynn, 1991). Specifically, a ‘commodity’ is valued in its level of unavailability. For example, Fromkin, Olson, Dipboye, and Barnaby (1971) found the rated value of a nylon hose was significantly higher if it was presented as having low availability (as opposed to high) and if participants thought they would be unable to take ownership of the hose. We note that this approach may be particularly relevant to the study of SSS because protocols tend to involve asking participants to evaluate both a food that is available to them eaten (the ‘eaten food’) and other foods that are not (the ‘uneaten food’) (e.g., Rolls et al., 1981).

The primary aim of this study was to investigate whether effects akin to those described by commodity theory can account for SSS. We reasoned that since the uneaten foods are unavailable, their relative value may increase. This causes a contrast with the eaten food, producing a relative decrease in its value. In other words, an eaten food becomes relatively less appealing *because* other unavailable uneaten foods increase in their value. In Study 1 we explored this idea by manipulating the perceived availability of uneaten foods. This between-subjects study comprised the following conditions; in the ‘available uneaten foods’ condition, the participants initially tasted and rated a set of foods. They were then told that they would be given one of these foods to eat *ad libitum* and that they could switch to another food afterwards. In an ‘unavailable uneaten foods’ condition, participants initially tasted and rated a set of foods and were then told they would receive one of the foods to eat *ad libitum* but that they would not be permitted to switch to a different food. Following commodity theory, it was hypothesised that greater SSS should be evident in the unavailable uneaten food condition compared to the available food condition.

The second aim of this study was to address a related and fundamental question about whether the mere presence of uneaten foods influences the evaluation of the eaten food (i.e., a participant is given a single food to taste and rate, and no mention is made about the uneaten foods or their availability). This question is important because a typical meal rarely involves uneaten foods that are tasted at the beginning of a meal and which become available upon meal termination. In keeping with commodity theory, we hypothesised that the decline in value of the eaten food (pleasantness and desire to eat) should be attenuated when no uneaten foods are present. This is because there are no unavailable comparators (uneaten foods) to generate a contrast effect. Therefore, an additional condition was added to the study, in which participants were presented with a single food to taste and rate, and consume as their lunch. This resulted in a study comprising three conditions, which together addressed both the primary and secondary aims.

## 2 Study 1

### 2.1 Method

#### 2.1.1 Participants

Sixty female staff and students from the University of Bristol (age, *M* = 26 years, *SD* = 10) participated in the study. The mean BMI was 23.4 kg/m^2^ (*SD* = 4.2). A between-subjects design was employed. Participants were randomly assigned to a condition on arrival. The protocols for studies 1–3 were approved by the local Faculty of Science Human Research Ethics Committee. Vegetarians, vegans and anybody with relevant food allergies were excluded from all three studies. Participants for all three studies were remunerated with course credit when they had completed their respective study.

#### 2.1.2 Rating scales

Following previous studies, we assessed the pleasantness and desire to eat a range of test foods (Rolls et al., 1992, Rolls and McDermott, 1991). In broad terms, these measures are thought to reflect two different aspects of palatability, wanting and liking of food (see Havermans, Janssen, Giesen, Roefs, & Jansen, 2009 for further discussion of these measures). Participants were asked to rate ‘How pleasant is the taste of this food right now?’ for each food on a 100 mm VAS, anchored ‘Not at all’ to the left and ‘Extremely’ to the right. Participants were also asked to rate ‘How strong is your desire to eat this food right now?’ for each food on a 100 mm VAS anchored ‘Not at all’ to the left and ‘Extremely’ to the right. These rating scales were implemented on a computer. The questions were always presented in the order described here. By contrast, the order in which the foods were rated was randomised by the computer; participants were told to either taste food 1, 2, 3 or 4 (foods were presented with corresponding numbers).

In addition, participants were asked to rate ‘How hungry are you now?’ and ‘How full are you right now’ on separate pen and paper 100 mm VAS anchored ‘Not at all’ to the left and ‘Extremely’ to the right.

#### 2.1.3 Foods

Consistent with the majority of studies exploring SSS (for a review see Sørensen, Møller, Flint, Martens, & Raben, 2003), participants were presented with food to eat *ad libitum*. Our own observations of *ad libitum* eating indicate that food intakes are often subject to extreme outliers. In particular, some men consume very large meals that are unlikely to reflect natural dietary behaviour. For this reason, we limited our sample to include only female volunteers.

Four commonly consumed lunchtime snack foods, which are known to be generally well liked from previous work in the Nutrition and Behaviour Unit, were used in this study (Table 1 contains full macronutrient information; supplementary file). Two savoury and two sweet foods were provided. The two savoury foods were cheese and crackers, and ready salted crisps. The two sweet foods were chocolate chip cookies and cake. All of the foods were produced and sourced from a well known supermarket in the UK (Sainsbury's Supermarkets Ltd, London). When the food was presented for tasting and rating, a bite-sized piece of each food was presented. When the food was provided as the eaten food, *ad libitum* access to the food was provided. The experimenter covertly recorded *ad libitum* intake by weighing (in grams) the food before and after consumption in a room separate to the participant testing area. Every food was presented as an eaten food and an uneaten food on the same number of occasions across participants. In so doing, the allocation of eaten and uneaten foods was fully counterbalanced across conditions.

#### 2.1.4 Procedure

Participants were asked to abstain from food for three hours prior to their testing session. All sessions lasted 60 min and took place between 11 a.m. and 2 p.m. Upon arrival, participants were provided with an information sheet to read and a consent form to sign. They then rated their hunger and fullness.

The procedure then differed depending on condition. In the ‘no uneaten foods condition’, participants were presented with a tray containing one bite-sized piece of food. The computer then instructed the participants to taste the food and then to rate its pleasantness and their desire to eat the food. The participants were then provided with *ad libitum* access to the food they had just tasted and rated. They were told that this was their lunch and that an additional serving of the food was available should they finish the plate that was in front of them (Table 1 contains quantities of foods that were presented to participants; supplementary file). Once the participants indicated that they had finished eating, they were asked to evaluate its palatability and their desire to eat once again.

In the ‘unavailable uneaten foods condition’, participants were given a tray containing numbered bite-sized pieces of four foods. The computer indicated which of the four foods to taste by number. They were then asked to rate the food for pleasantness and desire to eat. These ratings were completed for each of the four foods. Following this, the participants were provided with *ad libitum* access to one of the foods, given the same instruction as in the no uneaten foods condition but told that the other foods would not be available for their consumption during the study. At the end of the meal the participants were asked to evaluate all four foods using the two measures.

The ‘available uneaten foods condition’ followed the same protocol as the unavailable uneaten foods condition. However, when participants were given *ad libitum* access to one of the four foods that had been tasted and rated, they were told that they could switch to any of the other foods after they had finished consuming as much as they wanted. The final difference between this condition and the unavailable uneaten foods condition was that when participants had completed their final tastings and ratings of the four foods, they were provided with *ad libitum* access to the food that they had chosen as their ‘switch’ food.

Finally, all of the participants completed the Three Factor Eating Questionnaire (TFEQ; Stunkard & Messick, 1985). Their height and weight was recorded by the experimenter and a debrief sheet was provided.

#### 2.1.5 Data analysis

One-way ANOVAs were conducted to determine significant differences in baseline appetite ratings and individual differences across the samples allocated to each of the three conditions. For each food, change scores were then calculated; the baseline rating (pleasantness and desire to eat) was subtracted from the respective rating at meal termination. An average of the change scores relating to the uneaten foods was then calculated (for the available and unavailable variety conditions only). Specifically, for ratings of pleasantness and for ratings of desire to eat, a change score (pre-post eating) was calculated for the eaten food and a separate change score was calculated for the composite rating derived from the uneaten foods (Table 6 contains means and standard deviations of the absolute values used to calculate change scores; supplementary file). Thus, for each participant, 4 change scores were derived (2 measures; palatability/desire to eat) x 2 (food type; eaten/uneaten).

To assess the effect of SSS and the manipulation of perceived availability of the uneaten foods, a mixed ANOVA with food-type (eaten and average uneaten) as a within-subjects factor and condition (available and unavailable uneaten foods) as a between subjects factor was conducted on the change scores. This type of ANOVA was repeated for each of the dependent measures (pleasantness and desire to eat). Note that these measures were not taken in the no uneaten foods condition. Therefore data from this condition were not included in this analysis.

In order to address the second hypothesis which concerned the effect of the mere presence of uneaten foods on the change in ratings of the eaten food, a one-way ANOVA with condition (no uneaten foods, unavailable uneaten foods and available uneaten foods) as the between-subjects variable was conducted on the change in ratings from baseline to meal termination of the eaten food. This type of ANOVA was repeated for each of the measures (pleasantness and desire to eat).

### 2.2 Results

#### 2.2.1 Participant characteristics

There were no significant differences in initial hunger, initial fullness, restraint, disinhibition, age, BMI and *ad libitum* intake across conditions. See Table 2 (supplementary file) for associated statistics.

#### 2.2.2 SSS

Consistent with SSS, there was a significant difference in the change in pleasantness from baseline to meal termination between the eaten food (*M* = −24.7 mm, *SE* = 4.1) and the average of the uneaten foods (*M* = −3.8 mm, *SE* = 2.4; *F* (1,38) = 29.76, *p* < 0.001, η_p_^2^ = 0.41). There was also a significant difference in the change in desire to eat from baseline to meal termination between the eaten food and the average of the uneaten foods (eaten food, *M* = −32.3 mm, *SE* = 4.3; averaged uneaten foods *M* = −6.6 mm, *SE* = 3.3; *F* (1,38) = 39.38, *p* < 0.001, η_p_^2^ = 0.51).

#### 2.2.3 Manipulation of perceived availability of food

**Fig. 1:**
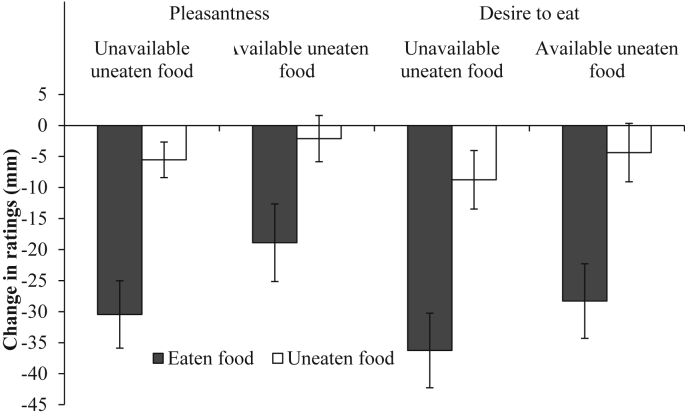
Mean (± *SE*) change in rated pleasantness and desire to eat from baseline to meal termination for the eaten and uneaten foods across the unavailable and available uneaten food conditions.

Inconsistent with a contrast effect, there was no significant interaction between condition (unavailable and available uneaten foods) and the type of food (eaten and average uneaten) for the change in pleasantness from baseline to meal termination (*F* (1, 38) = 1.13, *p* = 0.29, η_p_^2^ = 0.029; Fig. 1).

A similar pattern of results was found for the change in desire to eat from baseline to meal termination, there was no significant interaction between condition and food type (*F* (1,38) = 0.189, *p* = 0.67, η_p_^2^ = 0.005; Fig. 1).

#### 2.2.4 Does the mere presence of uneaten foods affect the change in ratings of the eaten food?

**Fig. 2:**
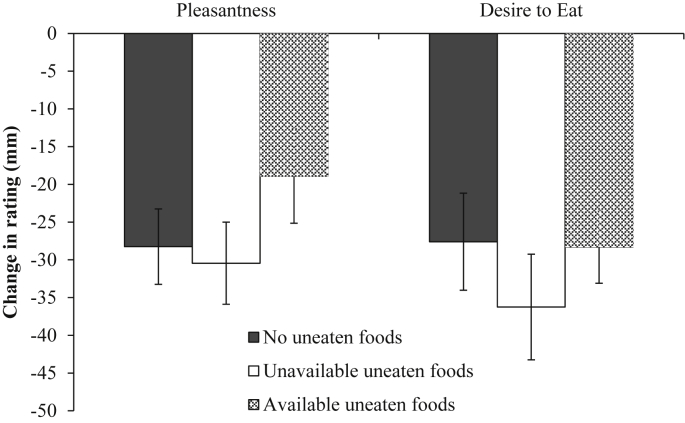
Mean (± *SE*) of the change in rated pleasantness and desire to eat of the eaten food from baseline to meal termination for the no uneaten foods, unavailable uneaten foods and available uneaten foods conditions.

The change in rated pleasantness of the eaten food (baseline compared to meal termination) did not differ significantly across the three conditions (no uneaten foods vs. unavailable uneaten foods vs. available uneaten foods) (*F* (2,57) = 1.2, *p* = 0.307, η_p_^2^ = 0.04). This was also the case for the change in rated desire to eat across conditions (*F* (2,57) = 0.61, *p* = 0.547, η_p_^2^ = 0.021). See Fig. 2 for the means and standard errors associated with the change in rated pleasantness and desire to eat across conditions.

### 2.3 Interim discussion

Our data indicate that the pleasantness for the eaten food decreased from baseline to a similar extent in both the unavailable and the available condition. Thus, we failed to garner evidence that the perceived availability of the uneaten foods (a contrast effect) can account for SSS. Nevertheless, the pattern of results was in the hypothesised direction.

The second aim of this study was to investigate the effect that the mere presence of uneaten foods might have on the evaluation of the eaten food. In a separate analysis, the decrement in pleasantness and desire to eat of the eaten food was compared across the no uneaten food condition and the available and unavailable uneaten food conditions. This showed that the eaten food was valued to a similar extent, irrespective of whether uneaten foods were present.

A potential concern is that our manipulation of food availability was not ‘believable’. In the available uneaten foods condition, participants were given verbal instructions informing them that they were free to switch to an alternative food after they had received *ad libitum* access to the first food. Therefore, in Study 2 we included additional measures to ensure that all participants were reminded of these instructions throughout.

A second concern is that participants were free to determine the size of their meal. This gave participants some control over the eating episode (the amount eaten). Previous research has suggested that a sense of control over the environment leads to a more positive evaluation of that environment (Proshansky, Ittelson, & Rivlin, 1974). One possibility is that this sense of control undermined the manipulation of perceived availability of food; while participants did not have control over the foods that they could eat in the unavailable uneaten foods condition, they did have control over the amount that they ate. Therefore, in Study 2 we incorporated a fixed portion of the eaten food.

## 3 Study 2

### 3.1 Method

#### 3.1.1 Participants

Sixty students (male *n* = 26) from the University of Bristol (age, *M* = 26 years, *SD* = 10) participated in the study. The mean BMI was 23.4 kg/m^2^ (*SD* = 3.7). Participants were assigned to a condition on arrival.

#### 3.1.2 Rating scales

The ratings scales used in this study were the same as those described in Study 1 with a number of additions. Participants were asked to complete three ratings relating to the size of the fixed portion of the eaten food that they received. Before consuming the food, they were asked ‘Is this portion too large or too small for you right now?’ Responses were made on a 100 mm VAS anchored to the left with ‘Too small’ and anchored to the right with ‘Too large’. After consuming the food, they were asked ‘Was that portion too much or too little food for you?’ Responses were made on a 100 mm VAS anchored to the left with ‘Too little’ and to the right with ‘Too much’. Finally, they were asked ‘To what extent could you eat that portion of food again?’ Participants were asked to respond on a 100-mm VAS anchored to the left with ‘None of it’ and to the right with ‘All of it’.

#### 3.1.3 Questionnaires

As in Study 1, participants were asked to complete the TFEQ (Stunkard & Messick, 1985). In addition, at the end of the study, participants were asked to answer two questions. Firstly, ‘What do you think this experiment was about?’ and secondly, ‘Did you believe what was said to you or did you have doubts at any time?’ A ‘free-writing’ space was provided underneath each question.

#### 3.1.4 Test foods

By contrast to Study 1, a fixed portion rather than an *ad libitum* portion was provided. Participants were asked to eat this portion in its entirety. This change obviated concerns about some male participants consuming portions that do not reflect natural dietary behaviour and therefore both male and female participants were recruited for this study. To simplify the design of this study, two foods were evaluated by participants. One of these foods became the eaten food whilst the other became the uneaten food. The order in which this occurred was fully counterbalanced across participants. Two commonly consumed savoury foods (cheese sandwiches and sausages; Sainsbury's Supermarkets Ltd, London) were used in this study (Table 3 contains information about macronutrient composition; supplementary file). The cheese sandwiches were served in a 110 g portion that was comprised of small triangles (eighths of a sandwich) and for the sausages the 190 g portion comprised of small pieces (halves of the sausages). Portion sizes were based on commonly consumed portions from previous studies conducted in the Nutrition and Behaviour Unit. The foods were presented in the same way for tasting and rating, one small triangle of cheese sandwich and half a sausage. Each food was presented as an eaten food and an uneaten food on the same number of occasions. In so doing, the allocation of eaten and uneaten foods was fully counterbalanced across participants.

#### 3.1.5 Procedure

With the following exceptions the protocol was the same as in Study 1. First, in the available condition when the eaten food was brought into the testing room for consumption by the participant, a portable ‘buffet-cart’ displaying a plate of the uneaten food was also brought into the room. This change was introduced in order to reinforce the verbal information given to the participant concerning the availability of the alternative food. Second, participants were provided with a fixed portion of food to consume rather than *ad libitum* access. They were told that this was their lunch and were asked to decide whether this portion was too large or too small. They were then asked to eat the portion in its entirety. Third, after consuming their fixed portion of food they were asked to evaluate the size of the portion that they had consumed. Finally, participants were asked to complete the brief ‘end of experiment’ questionnaire, as described above.

#### 3.1.6 Data analysis

One-way ANOVA was used to identify whether there were any significant differences in relevant measures of appetite and individual differences across conditions. Chi-square was used to identify whether there was a significant difference in sex across conditions. In addition, to assess whether the fixed portion was sufficient to significantly reduce hunger and to increase fullness, pre- and post-meal ratings of hunger and fullness were compared using paired-samples *t*-tests.

For each food, the change in pleasantness and desire to eat from baseline to meal termination was calculated; the baseline rating was subtracted from the respective rating at meal termination (Table 6 provides means and standard deviations of the absolute values used to calculate these change scores; supplementary file). This produced four change scores overall; change in pleasantness of the eaten food, change in pleasantness of the uneaten food, change in desire to eat of the eaten food and change in desire to eat the uneaten food.

To assess evidence for SSS and a contrast effect associated with the manipulation of perceived availability of the uneaten foods, a mixed ANOVA with food-type (eaten and average uneaten) as a within-subjects factor and condition (available and unavailable uneaten foods) as a between subjects factor was conducted on the change scores. This type of ANOVA was repeated for each of the dependent measures (pleasantness and desire to eat). Note that these measures were not taken in the no uneaten foods condition. Therefore data from this condition were not included in this analysis.

To assess the effect of the mere presence of uneaten foods on the change in ratings of the eaten food, a one-way ANOVA with condition (no uneaten foods, unavailable uneaten foods and available uneaten foods) as a between-subjects variable was conducted on the change in pleasantness from baseline to meal termination of the eaten food. The same analysis was repeated to evaluate ratings of desire to eat.

### 3.2 Results

#### 3.2.1 Participant characteristics

A comparison of age, BMI, eating time, restraint, disinhibition, hunger (TFEQ), initial hunger, initial fullness, post-meal hunger and fullness, pre- and post-meal portion size judgement, and a willingness to eat the portion again, revealed no significant differences between the groups (for descriptive statistics and associated statistics see Table 4 in the supplementary file). In addition, there was no significant difference in sex across conditions (20 participants were tested in each condition, of which 7 were male in the no uneaten foods condition, 12 were male in the unavailable uneaten foods condition, and 7 were male in the available uneaten foods condition; χ^2^ (2, *N* = 60) = 3.39, *p* = 0.18). Finally, all participants reported that they ‘believed’ that they would receive the food and the choices they were told they would receive and no participants guessed the aim of the study.

#### 3.2.2 Hunger and fullness

There was a significant reduction in reported hunger from pre- (*M* = 67.5 mm, *SE* = 2.4) to post-meal (*M* = 30.1 mm, *SE* = 2.9; *t* (59) = 11.61, *p* < 0.001). There was also a significant increase in reported fullness from pre- (*M* = 21.3 mm, *SE* = 2.6) to post-meal (*M* = 66.9 mm, *SE* = 2.9; *t* (59) = 13.74, *p* < 0.001). After participants had consumed their meal and completed necessary ratings/tasks, those in the available condition were given the opportunity to consume more of the eaten food and/or the uneaten food. Nine out of 20 participants chose to consume more food.

#### 3.2.3 SSS

Consistent with SSS, there was a significant difference in the change in pleasantness from baseline to meal termination between the eaten food (*M* = −19.4 mm, *SE* = 3.5) and the uneaten food (*M* = −0.4 mm, *SE* = 2.7; *F* (1,38) = 23.6, *p* < 0.001, η_p_^2^ = 0.384). There was also a significant difference in the change in desire to eat from baseline to meal termination between the eaten food and the average uneaten foods (eaten food, *M* = −34 mm, *SE* = 4; uneaten food, *M* = −15.3 mm, *SE* = 3.1; *F* (1,38) = 17.2, *p* < 0.001, η_p_^2^ = 0.312).

#### 3.2.4 Manipulation of perceived availability of food

**Fig. 3:**
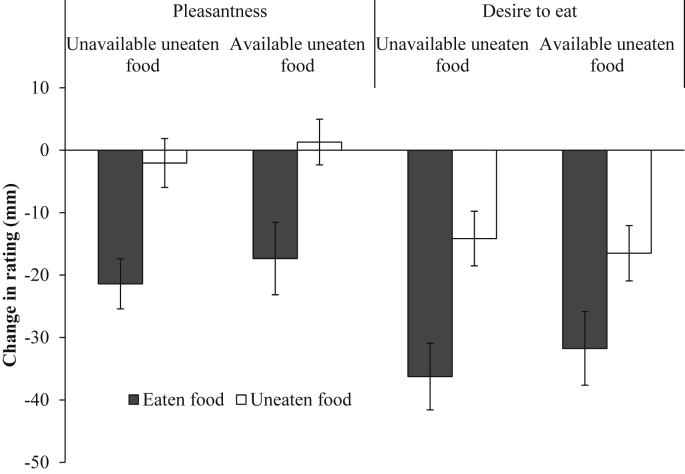
Mean (± *SE*) change in rated pleasantness and desire to eat from baseline to meal termination for the eaten and uneaten foods across the unavailable and available uneaten food conditions.

Inconsistent with a contrast effect, there was no significant interaction between condition (unavailable and available uneaten foods) and the type of food (eaten and average uneaten) for the change in pleasantness from baseline to meal termination (*F* (1, 38) = 0.008, *p* = 0.929, η_p_^2^ < 0.001; Fig. 3).

A similar pattern of results was found for the change in desire to eat from baseline to meal termination. There was no significant interaction between condition and food type (*F* (1,38) = 0.579, *p* = 0.45, η_p_^2^ = 0.015; Fig. 3).

#### 3.2.5 Does the mere presence of uneaten foods affect the change in ratings of the eaten food?

**Fig. 4:**
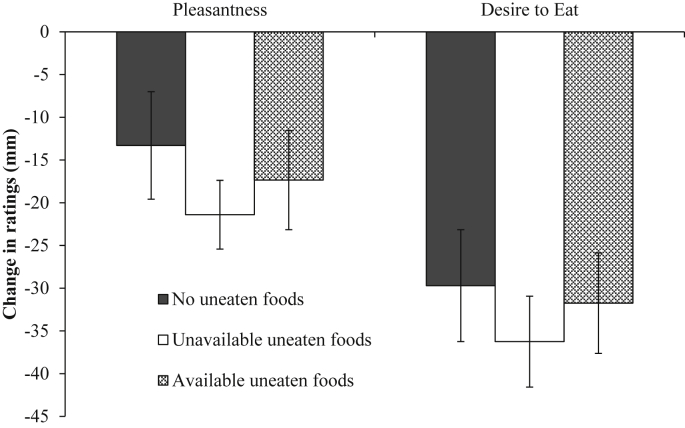
Mean (± *SE*) of the change in rated pleasantness and desire to eat of the eaten food from baseline to meal termination for the no uneaten foods, unavailable uneaten foods and available uneaten foods conditions.

There were no significant differences in the change in rated pleasantness from baseline to meal termination of the eaten food between the conditions (no uneaten foods, unavailable uneaten foods and available uneaten foods) (*F* (2,57) = 0.55, *p* = 0.58, η_p_^2^ = 0.019). This was also the case for the change in rated desire to eat across conditions (*F* (2,57) = 0.318, *p* = 0.729, η_p_^2^ = 0.011). See Fig. 4 for the means and standard errors associated with the change in rated pleasantness and desire to eat across conditions.

### 3.3 Interim discussion

The primary aim of this study was to improve upon the methodology presented in Study 1 in order to investigate whether the perceived availability of an uneaten food affects SSS. Following commodity theory, it was hypothesised that greater SSS should be evident in the unavailable uneaten food condition compared to the available food condition. However, there was little evidence to suggest that this was the case; there was no difference in the change in pleasantness and desire to eat of the eaten and uneaten foods across these conditions. This finding is consistent with Study 1.

As in Study 1, the second aim of this study was to investigate whether the mere presence of an uneaten food affected the evaluation of the eaten food. Consistent with Study 1, there was no significant difference in the evaluation of the eaten food across the no uneaten food and the available and unavailable uneaten food conditions. Broadly, this finding supports an explanation of SSS based on habituation or stimulus specificity because the presence or absence of uneaten foods had little effect on the observed within-meal decline in the value of the eaten food.

## 4 Study 3

### 4.1 Overview

Study 1 and Study 2 explored the prospect that the pattern of results observed in SSS studies is governed by beliefs about the availability of uneaten foods. Consistent with commodity theory, uneaten foods should increase in relative value at the point at which their unavailability is confirmed by the experimenter. In a standard SSS paradigm this tends to occur shortly before participants start to consume the eaten food. Accordingly, in Study 3 we tested the hypothesis that SSS (differences in pleasantness and desire to eat) might be evident even at the beginning of a meal, shortly after participants are informed which food is available and which food(s) are unavailable.

In order to isolate a contrast effect associated with perceived availability, an additional tasting and rating task was included before participants were offered the eaten food to consume for their lunch. According to an account based on a contrast effect, the eaten food should receive lower ratings, but only in the second set of ratings, after it becomes clear to the participants which foods will not be eaten. This could be attributed solely to a top-down contrast effect as both tasting and rating tasks will have been completed before consumption of the eaten food.

### 4.2 Method

#### 4.2.1 Participants

Forty-eight students (female = 38) from the University of Bristol (age, *M* = 22 years, *SD* = 8) assisted with the study. Their mean BMI was 21.8 kg/m^2^ (*SD* = 3.6).

#### 4.2.2 Procedure

All participants were asked to abstain from food for three hours prior to their testing session. Every session took place between 11am and 2pm and lasted up to 30 min. Upon arrival, participants were given an information sheet outlining the general protocol and a consent form to sign. They were then given an instruction sheet explaining how to use a VAS and asked to rate their current hunger and fullness level.

The participants were offered a sample of each of the four foods (numbered). The computer instructed them to taste each food (randomised order) and rate the food for pleasantness and desire to eat. After tasting each food, the experimenter prompted participants to take a sip of water. Following this, the participants were told which of the foods would be available to them to consume *ad libitum* and which would be unavailable. They were then asked to complete a dummy task (see below for a description of this task). A second set of hunger and fullness ratings was completed, followed by the tasting and rating of all four foods for a second time (again, pleasantness and desire to eat were rated).

Following this, the experimenter measured the participant's height and weight, and asked them to complete the TFEQ (Stunkard & Messick, 1985). Finally, the participants were told that they did not have to eat any more food (although food was provided upon request for ethical reasons) and told that the experiment had finished. They were given a debrief sheet to take away with them.

#### 4.2.3 Ratings

The ratings scales and the way they were administered was the same as in Studies 1 and 2.

#### 4.2.4 Dummy task

A dummy task was provided between the first and second sets of ratings. This was provided so that participants would be less likely to remember and recall the location of their first rating when making their second rating. This task involved participants looking at 25 logos associated with commonly consumed food products. They were asked to rate “How healthy do you consider the following product?” on a Likert scale anchored very unhealthy (1) to very healthy (7).

#### 4.2.5 Foods

Four commonly consumed lunchtime snack foods, which are known to be generally well liked from previous work in the Nutrition and Behaviour Unit, were used in the study (Table 5 contains full nutritional information; supplementary file). The two savoury foods used were ready salted crisps and wholemeal bread with cream cheese. The two sweet foods used were chocolate chip cookies and Madeira cake bar (Sainsbury's Supermarkets Ltd, London). When the food was presented for tasting and rating, a bite-sized piece of each food was presented. Every food was presented as an eaten food and an uneaten food on the same number of occasions. In so doing, the allocation of eaten and uneaten foods was fully counterbalanced across conditions. As in Study 2, both male and female participants were recruited as participants.

#### 4.2.6 Data analysis

Baseline ratings of pleasantness and desire to eat were subtracted from respective ratings at the second tasting and rating time (Table 6 contains means and standard deviations of the absolute values used to calculate change scores; supplementary file). For every participant and each food, this provided a change score for pleasantness and change score for desire to eat. Change scores for the uneaten foods were then averaged. Paired-samples *t*-tests were used to compare change scores for the eaten food and the uneaten foods. Separate tests were applied to scores relating to pleasantness and desire to eat.

### 4.3 Results

#### 4.3.1 Participant characteristics

The sample had a mean initial hunger rating of 64.9 mm (*SD* = 19.2) and a mean initial fullness rating of 19.5 mm (*SD* = 17.5). The sample had a mean restraint score of 8.6 (*SD* = 5.1), a mean disinhibition score of 7.7 (*SD* = 3.4), and a mean hunger score of 6.5 (*SD* = 3.8).

#### 4.3.2 Evidence for a contrast effect

**Fig. 5:**
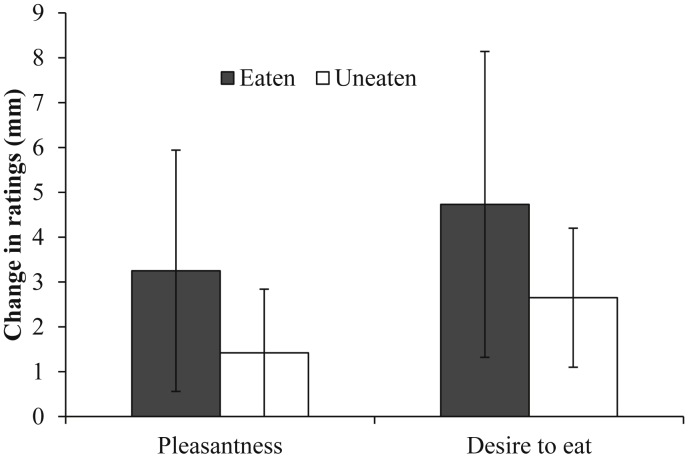
The mean and *SE* change in ratings from baseline for the eaten food and uneaten foods (averaged).

Inconsistent with a contrast effect, there was no significant difference between change scores for the eaten (*M* = 3.3 mm, *SE* = 2.7) and the uneaten (*M* = 1.4 mm, *SE* = 1.4) foods, both for pleasantness (*t* (47) = 0.73, *p* = 0.47, Cohen's *d* = 0.105) and desire to eat (eaten food, *M* = 4.7 mm, *SE* = 3.4; average uneaten food, *M* = 2.7 mm, *SE* = 1.6; *t* (47) = 0.72, *p* = 0.48, Cohen's *d* = 0.103; Fig. 5).

## 5 General discussion

The primary aim of Study 3 was to investigate whether a contrast effect based on the perceived availability of the eaten and uneaten foods was evident before participants began consuming the eaten food for their lunch. The results suggest that this was not the case; there was no significant difference in the change in pleasantness or desire to eat between the available (eaten) and unavailable (uneaten) foods. This result is consistent with the results of Study 1 and Study 2 which also failed to show any difference in decline in pleasantness or desire to eat based on our manipulation of perceived availability (following commodity theory). In addition, Study 1 and Study 2 also showed that the mere presence of uneaten foods did not affect the decline in pleasantness and desire to eat that is associated with the expression of SSS.

These results are consistent with those of Havermans and Brondel (2013) who also failed to find evidence for an effect of perceived variety on SSS. Havermans and Brondel (2013) suggest that this may be because they presented only a single comparator food, or because participants did not expect to receive the comparator food. Study 1 and Study 2 failed to support these hypotheses (respectively, Study 1 and Study 2 addressed the former and the latter). Taken together, these findings support an explanation for SSS based on habituation (Higgs et al., 2008) or stimulus specificity (Havermans, 2012). Nevertheless, if SSS is ‘just’ an expression of habituation or stimulus specificity, why does changing the colour of chocolates (Rolls et al., 1982) or manipulating participants' perception of flavour variety (Redden, 2008) affect satiation?

One possibility is that these findings reflect anticipatory processes. Wilkinson, Hinton, Fay, Rogers, and Brunstrom (2013) demonstrated that the ‘variety effect’ (closely related to SSS; Brondel et al., 2009b, Brondel et al., 2009a) is anticipated in meal planning. When presented with photographs of two course meals, ideal portion size and rated pleasantness of the second course increased as the difference in sensory characteristics increased across the courses. This finding was replicated in the context of snack foods (photographs of two plates of snack foods displayed side-by-side that were either similar or different in their sensory characteristics). The anticipation of the variety effect is likely to be based on our memory for the consumption of meals and snacks that vary in sensory characteristics. Such anticipatory effects may act to interfere with or bias the experience of habituation (for further discussion on the topic of memory and appetite see Brunstrom et al., 2012).

Also noteworthy in this context are results showing that merely imagining eating a food (repeatedly) led to a reduction in the subsequent actual consumption of that food compared to food where there had been no imagined consumption or consumption of the food had been imagined fewer times (Morewedge, Huh, & Vosgerau, 2010). Morewedge and colleagues suggest that imagining a food repeatedly ‘engendered’ habituation.

Both of these examples (Morewedge et al., 2010, Wilkinson et al., 2013), which require some component of top-down processing (e.g., memory for eating a varied meal or a mental representation supporting the imagined consumption of food, respectively), are closely tied to the basic expression of SSS. By contrast, the studies presented here, which failed to show any top-down influence on SSS, related to participants’ perception of the availability of the uneaten foods.

We also note consistencies between the three studies reported here and those from related fields. For example, Werthmann, Roefs, Nederkoorn, and Jansen (2013) failed to find effects of perceived availability (of chocolate) on craving, attentional bias or food intake. In another context, Carter and Tiffany (2001) found that perceived availability and the expectation of receiving a cigarette (as opposed to the unavailability of items that is the focus of commodity theory) was associated with increases in craving, attentional bias and drug seeking.

Whilst these studies did not yield significant results, they systematically tested hypotheses focused on the protocol that tends to be used in SSS studies, within a well-established theoretical framework (commodity theory). They extended results presented previously (Havermans & Brondel, 2013), showing that the perception of availability of uneaten foods and the mere presence of uneaten foods does not affect the expression of SSS. Broadly, they support an explanation of SSS based on habituation or stimulus specificity rather than top-down influences based on the availability of uneaten foods.
