# Supplementary material for: Sensory specific satiety: More than ‘just’ habituation?
Source: Appetite. 2016 Aug 1;103:221–8. doi: 10.1016/j.appet.2016.04.019 (PMC4910838; doi:10.1016/j.appet.2016.04.019)
Supplement: Supplementary file 1 [file mmc1.docx]

Table 1. The macronutrient composition of each of the test foods and amount presented for *ad libitum* consumption in Study 1.

|  | Kcal/ 100 g | Protein/  100 g | Carbohydrate/ 100 g | Fat/ 100 g | Amount presented (g) |
| --- | --- | --- | --- | --- | --- |
| Cheese & Crackers | 409 | 16.4 | 38 | 19.3 | 1150 |
| Crisps | 526 | 5.1 | 52.8 | 32.7 | 750 |
| Cookies | 496 | 5.9 | 64.3 | 23.9 | 950 |
| Cake | 293 | 3.4 | 64.8 | 2.2 | 850 |

Table 2. For each participant characteristic, means and standard error for each condition as well as the *F* and *p* values associated with the one-way ANOVA (*df* =2) comparing these conditions in Study 1.

|  | No uneaten foods  (*n* = 20) | | Unavailable uneaten foods  (*n* = 20) | | Available uneaten foods  (*n* = 20) | | *F* | *p* |
| --- | --- | --- | --- | --- | --- | --- | --- | --- |
|  | *M* | *SE* | *M* | *SE* | *M* | *SE* |  |  |
| Initial hunger (mm) | 65.7 | 4.4 | 76 | 2.4 | 63 | 3.9 | 2.2 | .12 |
| Initial fullness (mm) | 15.9 | 2.7 | 13.7 | 1.7 | 23.4 | 4.4 | 2.6 | .08 |
| Restraint | 7.5 | 1.2 | 9.1 | 1.2 | 8 | 1.2 | .4 | .66 |
| Disinhibition | 8.3 | .8 | 6.3 | .7 | 6.8 | .6 | .4 | .14 |
| Age (years) | 22.9 | 1.2 | 29.9 | 2.8 | 25.9 | 2.4 | 2.5 | .09 |
| BMI (kg/m^2^) | 24.6 | 1.3 | 23.3 | .5 | 22.4 | .7 | 1.4 | .27 |
| Intake (kcal) | 427.8 | 58.5 | 374.2 | 39.6 | 315.7 | 38.8 | 1.5 | .24 |

Table 3. Macronutrient composition of the test foods in Study 2.

|  | Kcal/100g | Protein/100g | Carbohydrate/  100g | Fat/100g |
| --- | --- | --- | --- | --- |
| Cheese sandwich | 450 | 25.6 | 45.4 | 35 |
| Sausages | 242 | 13.2 | 14.2 | 14.7 |

Table 4. For Study 2, the mean and standard error for each participant characteristic within each condition and the *F-* and *p*-values associated with each one-way ANOVA (*df* = 2) conducted.

|  | No uneaten foods  (*n* = 20) | | Unavailable uneaten foods  (*n* = 20) | | | Available uneaten foods  (*n* = 20) | | |  | | |
| --- | --- | --- | --- | --- | --- | --- | --- | --- | --- | --- | --- |
|  | *M* | *SE* | | *M* | *SE* | | *M* | *SE* | | *F* | *p* |
| Age (years) | 27 | 2.6 | | 25 | 2.1 | | 25 | 2.2 | | .4 | .7 |
| BMI (kg/m^2^) | 23.3 | .98 | | 23.6 | .7 | | 23.3 | .8 | | .04 | .97 |
| Eating time (minutes) | 7.1 | .4 | | 6.1 | .5 | | 6.6 | .5 | | 1 | .36 |
| Restraint | 6.9 | 1.3 | | 5.2 | 1.1 | | 6.9 | 1.1 | | .7 | .52 |
| Disinhibition | 6.6 | .9 | | 6.3 | .8 | | 7.3 | 1 | | .3 | .74 |
| Hunger (TFEQ) | 6.9 | .7 | | 7.7 | .7 | | 6.1 | .8 | | 1.2 | .31 |
| Initial hunger (mm) | 63.8 | 4.4 | | 73 | 3.8 | | 65.8 | 4.3 | | 1.4 | .27 |
| Initial fullness (mm) | 22.5 | 5.1 | | 17.2 | 3.8 | | 24.3 | 4.7 | | .7 | .52 |
| Post-meal hunger (mm) | 27 | 4.6 | | 34.2 | 5.9 | | 29 | 4.4 | | .6 | .58 |
| Post-meal fullness (mm) | 68.2 | 4.2 | | 65.4 | 5.8 | | 67.1 | 5 | | .1 | .93 |
| Pre-meal portion size judgement (mm) | 58.6 | 4.5 | | 55 | 5.1 | | 47.5 | 4.7 | | 1.4 | .25 |
| Post-meal portion size judgement (mm) | 62.1 | 5.2 | | 55.5 | 5.3 | | 56.6 | 6 | | .4 | .67 |
| ‘Eat portion again’ judgement (mm) | 36.8 | 7.6 | | 30.3 | 7.2 | | 28.9 | 6.5 | | .4 | .71 |

Table 5. The macronutrient composition of each of the test foods in Study 3.

|  | Kcal/ 100g | Protein/ 100g | Carbohydrate/ 100g | Fat/ 100g |
| --- | --- | --- | --- | --- |
| Cookie | 469 | 5.9 | 64.3 | 23.9 |
| Cake Bar | 450 | 5.3 | 51.3 | 24.8 |
| Ready salted crisps | 526 | 5.1 | 52.8 | 32.7 |
| Wholemeal bread and cream cheese | 475 | 15.3 | 42.5 | 27.1 |

Table 6. The mean and standard deviation of absolute pleasantness and desire to eat ratings for the eaten and uneaten foods (averaged) at meal initiation and termination, shown for each condition of each study.

|  |  | Rated pleasantness | | Rated desire to eat | |
| --- | --- | --- | --- | --- | --- |
| **Study 1** |  | *M* (mm) | *SD* | *M* (mm) | *SD* |
| No uneaten foods | Baseline Eaten Food | 69.4 | 18.8 | 66.5 | 23 |
|  | Baseline Uneaten Food | N/A | | N/A | |
|  | Meal Termination Eaten Food | 41.1 | 18.1 | 27.9 | 16.4 |
|  | Meal termination uneaten food | N/A | | N/A | |
| Unavailable uneaten foods | Baseline Eaten Food | 66.7 | 14.4 | 64.6 | 22.5 |
|  | Baseline Uneaten Food | 64.6 | 11.1 | 61.1 | 15.4 |
|  | Meal Termination Eaten Food | 36.3 | 21.5 | 21.8 | 25.5 |
|  | Meal termination uneaten food | 59 | 18.5 | 52.4 | 20.3 |
| Available uneaten foods | Baseline Eaten Food | 61.5 | 20.4 | 56.3 | 18.8 |
|  | Baseline Uneaten Food | 66.5 | 11.7 | 56.7 | 13.6 |
|  | Meal Termination Eaten Food | 42.8 | 23.7 | 20.2 | 14.6 |
|  | Meal termination uneaten food | 64.3 | 11.5 | 52.3 | 19.2 |
| **Study 2** |  |  | |  | |
| No uneaten foods | Baseline Eaten Food | 63 | 20.8 | 58.2 | 24.3 |
|  | Baseline Uneaten Food | N/A | | N/A | |
|  | Meal Termination Eaten Food | 49.7 | 22.4 | 28.5 | 23.5 |
|  | Meal termination uneaten food | N/A | | N/A | |
| Unavailable uneaten foods | Baseline Eaten Food | 66.5 | 16.5 | 68.2 | 19.4 |
|  | Baseline Uneaten Food | 57.5 | 20.5 | 59.5 | 26.2 |
|  | Meal Termination Eaten Food | 45.1 | 22.5 | 31.1 | 31.2 |
|  | Meal termination uneaten food | 55.4 | 27.1 | 45.4 | 28.2 |
| Available uneaten foods | Baseline Eaten Food | 55.7 | 21.2 | 56.7 | 25.9 |
|  | Baseline Uneaten Food | 60.5 | 22.3 | 31.3 | 21.5 |
|  | Meal Termination Eaten Food | 38.3 | 24.6 | 24.9 | 23.1 |
|  | Meal termination uneaten food | 61.8 | 14.6 | 44.8 | 19 |
| **Study 3** |  |  | |  | |
|  | Baseline Eaten Food | 59.4 | 23.2 | 51.6 | 26.9 |
|  | Baseline Uneaten Food | 60.3 | 15.9 | 52.9 | 19.1 |
|  | Meal Termination Eaten Food | 62.6 | 18.2 | 56.3 | 22.1 |
|  | Meal termination uneaten food | 61.7 | 16.3 | 55.6 | 19.8 |
